# 

**Published:** 1881-12

**Authors:** 


### CONTENTS.

### COMMUNICATIONS.

Treatment of Pulpless Teeth and Manner of Filling Roots............ 447
Operative Dentistry............ 489
The Treatment of Pulpless Teeth............ 491
The Merits of Soft and Cohesive Gold as Filling Materials............ 493
What Ails the Dental Societies?............ 497

### SELECTIONS.

Medical Students............ 499
Our Medical Literature............ 503
Rodent Ulcer and Epithelioma............ 511
Practical Hints on Mounting............ 512
The Compensation of Expert Witnesses............ 513
Deaths from Ether............ 514
Fatal Poisoning by Inhalation of Carbolic Acid............ 515
Hints for Using Iodoform............ 515
The Ventilation of Bed Rooms............ 516
Treatment of Nasal Catarrh............ 517
Action Upon the Fœtus of Medicine taken by the Mother............ 518
Claims of the Microscope............ 519
The Care of the Dentists' Hands............ 520
The Hippocratic Oath............ 521
Curious Sanitary Discoveries............ 522
Mouth Wash for Tobacco Consumers............ 523

### EDITORIAL.

Engagement Books............ 523
The Physicians' Visiting List............ 523
The Dental Law in Ohio............ 524
Ohio State Society............ 524
Universal Rubber Dam Screw-Clamp............ 525
Rubber Dam Holder............ 526
Removal............ 526

